# Multi-omics perspectives on mitochondrial vulnerability in the aging liver: biological modules and translational implications

**DOI:** 10.3389/fcell.2026.1954948

**Published:** 2026-10-15

**Authors:** Hanxi Chen, Weiwei Lu, Kainan Wang, Rui Fang, Sunjae Lee, Shi Chen, Zhengtao Liu

**Affiliations:** 1 School of Medicine, Zhejiang Chinese Medical University, Hangzhou, China; 2 Key Laboratory of Artificial Organs and Computational Medicine in Zhejiang Province, Shulan International Medical College, Zhejiang Shuren University, Hangzhou, Zhejiang, China; 3 Department of General Practice, Shulan (Hangzhou) Hospital, Shulan International Medical College, Zhejiang Shuren University, Hangzhou, China; 4 Department of Hepatobiliary Surgery, Shulan (Hangzhou) Hospital, Shulan International Medical College, Zhejiang Shuren University, Hangzhou, China; 5 Graduate School of Engineering Biology, Korea Advanced Institute of Science and Technology, Daejeon, Republic of Korea; 6 Key Laboratory of Organ Transplantation, First Affiliated Hospital, School of Medicine, Zhejiang University, Hangzhou, Zhejiang, China

**Keywords:** biological modules, liver aging, machine perfusion, mitochondrial metabolism, mitochondrial vulnerability, multi-omics, oxidative stress, transplantation

## Abstract

Chronological age alone is an incomplete proxy for the biological heterogeneity of the aging liver. Mitochondrial vulnerability may provide a mechanistic link between biological aging and the loss of hepatic resilience under metabolic, toxic, and ischemic stress. Accumulating multi-omics evidence suggests that hepatic aging is best understood as a set of coordinated pathway-level disturbances rather than through isolated biomarkers. Transcriptomic, proteomic, and metabolomic studies converge on five interrelated biological modules: lipid stress, diminished oxidative reserve, proteostatic strain, inflammatory sensitization, and reduced recovery capacity. These modules are interconnected through impaired mitochondrial metabolic flexibility, redox buffering, organelle crosstalk, and quality-control mechanisms. Age-associated changes may remain partly compensated at baseline but become more apparent during metabolic overload, ischemia–reperfusion injury, cold preservation, or *ex vivo* machine perfusion. Building on cross-species evidence, this Review organizes current findings into a modular framework and examines how mitochondrial vulnerability may connect physiological liver aging with metabolic and ischemic stress. Transplantation and machine perfusion are discussed as stress-exposed settings for evaluating candidate tissue- and fluid-based readouts. Although most omics-derived signals remain insufficiently validated for clinical decision-making, a mitochondrial-centered interpretation may help prioritize biologically coherent mechanisms and improve the translational study of aging-related liver vulnerability.

## 1 Introduction

The liver is highly dependent on mitochondrial function to sustain fatty-acid oxidation, oxidative phosphorylation, redox homeostasis, detoxification, and regenerative activity (Hunt et al., 2019). During aging, these processes may become progressively less adaptable even when conventional histology and routine biochemical measurements remain relatively preserved. Declining mitochondrial metabolic flexibility, impaired quality control, and reduced antioxidant reserve may therefore represent an important biological substrate of age-related hepatic vulnerability (Castro et al., 2012; Niemann et al., 2017). Such changes are unlikely to be specific to transplantation and may also influence the response of older livers to metabolic overload, ischemia–reperfusion injury, and other acute challenges. Transplantation and machine perfusion are particularly informative because they impose defined metabolic and ischemic stress that may expose otherwise compensated mitochondrial dysfunction (Schlegel et al., 2020; Bhat et al., 2021; Hautz et al., 2024).

This aging-related vulnerability has particular clinical relevance in liver transplantation, where the global shortage of grafts has driven growing reliance on extended-criteria and older donors (Durand et al., 2019; Kwong et al., 2025). As waitlist pressures intensify, the clinical use of older grafts has become routine, yet post-transplant outcomes remain sensitive to donor age. A recent registry-based analysis of over 111,000 liver transplants reported that significant donor-recipient age mismatches—specifically when older grafts are allocated to younger recipients—were associated with lower 5-year graft survival (69.9%) compared with young-to-old combinations (77.8%) (Shirini et al., 2026). Grafts from donors over 60–70 years also fare poorly when allocated to high-risk recipients, such as those with MELD scores ≥35 (Shirini et al., 2026). Similar age-related declines in resilience have been documented even in living donor liver transplantation, where using donors aged 50 and older is associated with a 61% increased hazard of graft failure compared with younger cohorts (Kanneganti et al., 2023). Despite decades of relying on chronological age to assess liver graft suitability, clinicians have long recognized a clinical paradox: donor age alone incompletely predicts post-transplant behavior (Hunt et al., 2019; Allaire and Gilgenkrantz, 2020). Liver aging is therefore better viewed as a biologically heterogeneous state driven by more than chronological time (Morsiani et al., 2019; Wang et al., 2024b). Although some older livers retain substantial reserve, others are more susceptible to metabolic stress, inflammatory injury, and impaired recovery. Conventional descriptors, including donor age, histology, and routine biochemical tests, remain clinically useful, but they do not fully account for this variation. Instead, aging in the liver appears to reflect distributed changes in metabolic homeostasis and redox control, alongside shifts in proteostatic capacity, inflammatory tone, and recovery potential. These differences become especially relevant in transplantation, where predictable insults can convert modest baseline variation into clinically relevant differences in early graft function (Durand et al., 2019; Morsiani et al., 2019; Wang et al., 2024b).

This complexity is easily underestimated because the human liver often preserves architecture and functional reserve well into later life. At the molecular level, however, aging is associated with transcriptomic reorganization, altered chromatin accessibility and polymerase pausing, protein-level remodeling, and shifts in lipid and redox metabolism, even when histological change remains subtle (Bacalini et al., 2019; Schreiter et al., 2021; Bozukova et al., 2022; Ding et al., 2025; Sinha et al., 2026). In practice, these alterations often become most apparent when hepatic reserve is challenged by ischemia–reperfusion, preservation injury, or *ex vivo* perfusion (Schlegel et al., 2020; Bhat et al., 2021; Hautz et al., 2024).

Recent work has clarified this pattern. Oxidized lipid mediators such as 13-HODE have been linked to age-related steatosis and impaired catalase activity (Duan et al., 2023), dysregulated Hedgehog signaling has been implicated in reduced recovery capacity (Maeso-Díaz et al., 2022), and FGF21 and FABP4 have emerged as candidate nodes in aging-sensitive metabolic regulation (Lv et al., 2023; Wang et al., 2024a). Human and translational studies likewise suggest that vulnerability is amplified by altered intercellular signaling, exemplified by the liver sinusoidal endothelial cell–hepatocyte IL-1α–IL1R1 axis under ischemia–reperfusion stress (Liu et al., 2026). These findings shift the focus from discovering a single decisive biomarker toward mapping a set of interrelated disturbances, which are developed further in Section 4.

The evidence base, however, remains uneven. Rodent studies provide much of the current mechanistic depth (Liu et al., 2022; Williams et al., 2022; Tang et al., 2023; Mohallem et al., 2025), while non-human primate data improve biological plausibility yet remain limited in scale (Castro et al., 2012; Puppala et al., 2023; Yang et al., 2024). Spatial transcriptomic studies across aging tissues are also providing new perspectives; for example, immunoglobulin-associated senescence has been mapped as a spatial feature of multi-tissue aging, including liver-related changes (Ma et al., 2024). Transplant stress biology and baseline human aging datasets therefore remain sparse, and the bridge to clinical translation is still underdeveloped (Bhat et al., 2021; Schreiter et al., 2021; Hautz et al., 2024).

This review evaluates transcriptomic, proteomic, and metabolomic studies of liver aging, emphasizing how these layers converge on major biological modules. We focus on these omics layers because they are most directly linked to dynamic regulation, functional effectors, and physiological output, while epigenetic findings are cited primarily where they clarify upstream context. Following an analytical framework for interpreting omics findings across baseline and stress-exposed settings, we synthesize evidence spanning rodent to human models. We also explore transplantation and machine perfusion as clinical contexts in which aging-related vulnerability becomes more visible, while maintaining a clear distinction between mechanistic plausibility and clinical readiness. Unlike prior reviews that focus mainly on cellular senescence or general liver aging, this review organizes aging-related omics evidence around five biological modules and asks how these modules become visible in transplantation and machine perfusion settings.

## 2 Analytical framework for studying liver aging across omics layers

Molecular signals of liver aging depend on the biological setting. Three contexts are distinguished here: physiological baseline aging, acute challenge, and controlled *ex vivo* stress exposure. Baseline studies define age-associated change without imposed perturbation; ischemia–reperfusion, resection, inflammatory challenge, and metabolic overload reveal latent vulnerability; and machine perfusion provides a standardized interval in which tissue, perfusate, and bile can be sampled serially (Bacalini et al., 2019; Hunt et al., 2019; Allaire and Gilgenkrantz, 2020; Maeso-Díaz et al., 2022; Hautz et al., 2024; Eden et al., 2025).

Each context answers a different question. Baseline studies describe age-associated state, challenge models test stress responsiveness, and perfusion studies assess clinically accessible responses that may not be aging-specific. Figure 1 separates these contexts from omics layers, biological modules, and transplant-facing validation.

**FIGURE 1:**
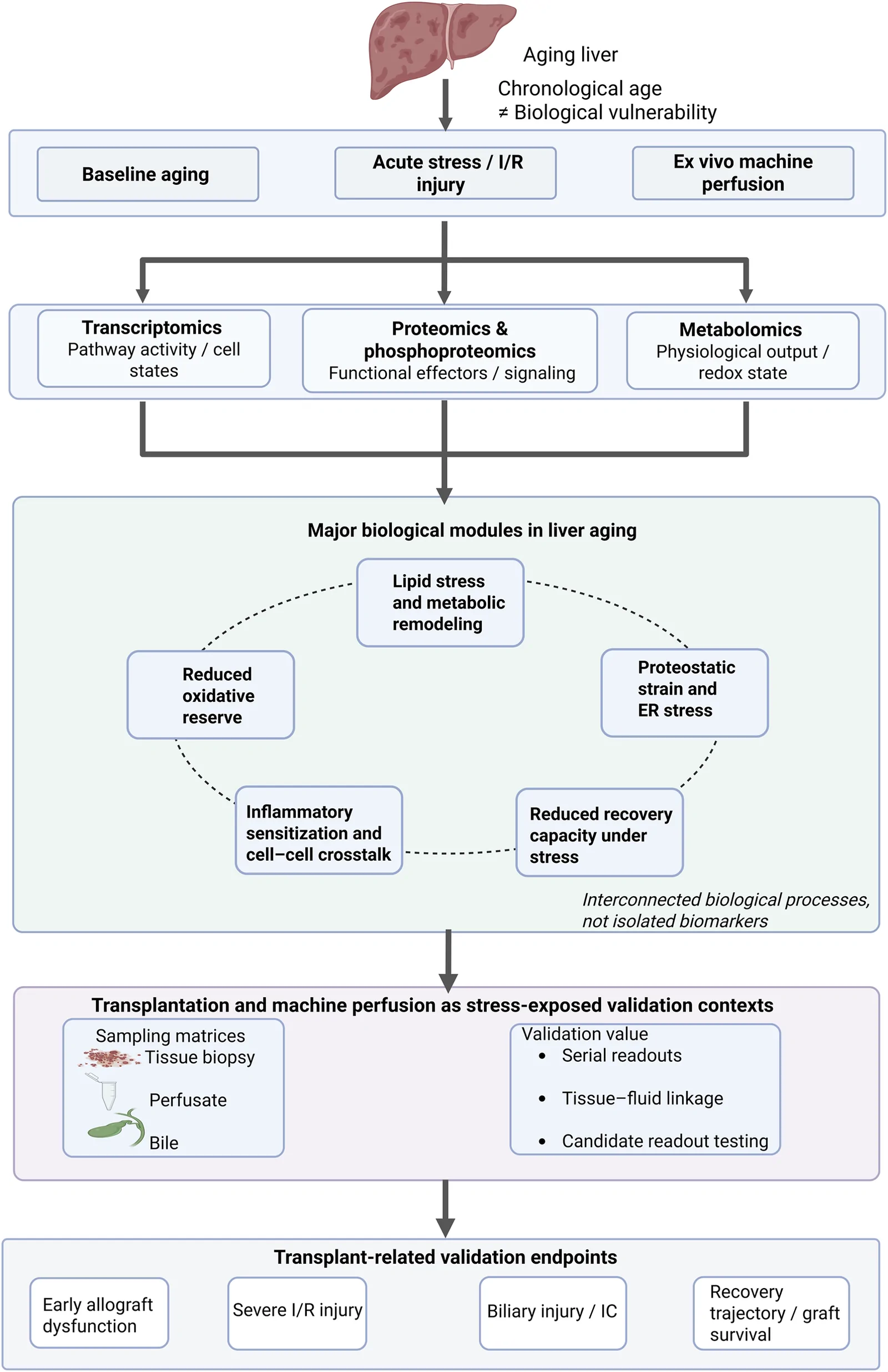
Multi-omics framework for interpreting aging-related liver vulnerability. This figure summarizes how liver aging can be interpreted across biological contexts, omics layers, major biological modules, and transplant-related validation settings. Baseline aging, acute stress or ischemia–reperfusion injury, and *ex vivo* machine perfusion represent distinct but related biological windows. Transcriptomic, proteomic/phosphoproteomic, and metabolomic evidence is organized around the five biological modules discussed in this review: lipid stress and metabolic remodeling, reduced oxidative reserve, proteostatic strain and ER stress, inflammatory sensitization and cell–cell crosstalk, and reduced recovery capacity under stress. Transplantation and machine perfusion provide stress-exposed settings in which tissue biopsy, perfusate, and bile may support serial readouts, tissue–fluid linkage, and candidate readout testing. The framework is intended to clarify biological interpretation and translational logic, rather than to represent a decision-ready clinical prediction model. Mitochondrial vulnerability is interpreted as a connecting mechanism across the five modules rather than as a separate biological module.

### 2.1 Baseline aging, stress models, and translational sampling settings

Baseline studies define gradual changes in relatively unperturbed liver, including altered zonation, chromatin accessibility, proteostatic burden, and metabolite composition (Bacalini et al., 2019; Schreiter et al., 2021; Bozukova et al., 2022; Nikopoulou et al., 2023; Sinha et al., 2026). Their main limitation is that clinically important vulnerability may remain buffered or weakly visible at rest (Hunt et al., 2019; Allaire and Gilgenkrantz, 2020).

Challenge models test reserve. Age-related differences in regeneration, inflammatory amplification, redox buffering, and recovery become more evident after ischemia–reperfusion, resection, or metabolic stress. These models provide mechanistic leverage, but general injury responses should not be treated as definitions of aging itself (Hunt et al., 2019; Maeso-Díaz et al., 2022; Wang et al., 2023).

Machine perfusion offers a translational setting for serial sampling of tissue, perfusate, and bile during a partly standardized stress interval. The resulting signals reflect donor comorbidity, ischemia, procurement, and preservation strategy as well as age-related vulnerability (Schlegel et al., 2020; Melandro et al., 2022). The contexts and matrices used in this review are summarized in Table 1.

**TABLE 1:** Biological contexts and sampling matrices for omics studies of liver aging and transplant stress.

Context or matrix	Material/readout	Best suited for	Strength	Boundary
Baseline liver	Biopsy, resection, sorted cells; tissue omics	Physiological aging substrate	Defines steady-state pathway change	May miss latent vulnerability
*In vivo* challenge	Tissue after I/R, resection, or metabolic stress	Reserve, inflammation, regeneration	Supports causal experiments	General injury may be mistaken for aging
Blood/plasma	Proteins, cfRNA, EVs, metabolites	Circulating surrogates	Scalable and longitudinal	Dilution and extrahepatic confounding
Perfusate	Serial HOPE/NMP proteins, enzymes, metabolites	Injury and recovery trajectories	Links fluid kinetics to tissue and outcomes	Protocol, ischemia, and donor effects
Perfusion bile	pH, bicarbonate, glucose, lipids, injury markers	Biliary viability	Organ-specific cholangiocyte window	Requires adequate bile production
Spatial liver sections	Spatial transcriptomics/metabolomics	Zonation and niche crosstalk	Preserves microenvironment	Cost, complexity, small cohorts

This table summarizes context-dependent omics sampling in liver aging. Different biological windows capture different aspects of hepatic biology, from steady-state molecular reorganization to stress-exposed vulnerability and clinically accessible fluid readouts. Findings from one context should not be directly generalized to another without validation.

Abbreviations: cfRNA, cell-free RNA; EVs, extracellular vesicles; HOPE, hypothermic oxygenated perfusion; I/R, ischemia–reperfusion; MALDI-MSI, matrix-assisted laser desorption/ionization mass spectrometry imaging; NMP, normothermic machine perfusion.

### 2.2 Why transcriptomics, proteomics, and metabolomics are complementary

Transcriptomics resolves pathway activity, cell-state transitions, zonation, and intercellular signaling. Single-cell and spatial approaches can localize age-associated changes in endothelial-hepatocyte communication and distinguish parenchymal from non-parenchymal responses (Nikopoulou et al., 2023; Lin et al., 2024; Tak et al., 2025; Sinha et al., 2026). These data identify regulatory programs, but do not by themselves establish pathway execution.

Proteomics and phosphoproteomics move closer to functional effectors by measuring protein abundance, signaling state, extracellular-matrix remodeling, and protein-maintenance pathways. Age-associated changes in Lumican and MAPK-related phosphorylation illustrate how protein-level data can refine or contradict transcript-based inference (Liu et al., 2022; 2025; Ding et al., 2025; Mohallem et al., 2025).

Metabolomics captures dynamic pathway output, including lipid handling, energy balance, and redox state (Duan et al., 2023; Tang et al., 2023; Wang et al., 2024a). Because metabolites are sensitive to diet, circadian timing, ischemia, perfusion, and sample handling, they are most informative when interpreted with transcriptomic, proteomic, or functional evidence rather than as isolated classifiers (Bhat et al., 2021; Buijink et al., 2024).

Together, the three layers provide complementary views of mitochondrial state: transcripts indicate regulatory programs, proteins and phosphoproteins indicate pathway execution, and metabolites indicate physiological output (Williams et al., 2022; Nikopoulou et al., 2023). None alone establishes mitochondrial dysfunction. Confidence is strongest when findings converge across layers and, where feasible, are supported by functional assays (Argelaguet et al., 2020; Subramanian et al., 2020).

### 2.3 From isolated markers to mitochondrial vulnerability modules

The literature contains many plausible candidates but few reproducible single markers. This is consistent with a biology that is distributed across pathways and cell states and is often exposed more clearly under challenge than at rest. A marker-first synthesis can therefore obscure process-level agreement.

Here, a biological module denotes a coherent pathway-level disturbance supported across omics layers, model systems, or contexts. Modules accommodate variation in individual transcripts, proteins, and metabolites while preserving continuity at the process level. Section 4; Table 2 therefore organize evidence around five vulnerability modules rather than an exhaustive marker inventory (Williams et al., 2022; Nikopoulou et al., 2023; Ding et al., 2025).

**TABLE 2:** Cross-omics evidence for major biological modules in liver aging.

Biological module	Transcript-level evidence	Protein/functional evidence	Metabolic readouts	Dominant cells/niches	Interpretive value
Lipid stress and metabolic remodeling	FAO, peroxisomal, FGF21/FABP4, SREBP2 programs	Lipid-handling enzymes; mitochondrial-ER contact signaling	Acylcarnitines, lipid mediators, 13-HODE	Hepatocytes; zonation-dependent	Reduced metabolic flexibility (Williams et al., 2022; Duan et al., 2023; Wang et al., 2024a)
Reduced oxidative reserve	OXPHOS, antioxidant, mitophagy programs	Catalase/SOD systems; respiratory-chain proteins	GSH/GSSG, ROS/RNS, MDA, lactate, FMN	Hepatocytes; endothelial and immune modifiers	Limited redox buffering under challenge (Castro et al., 2012; Bárcena et al., 2021; Huang et al., 2021)
Proteostatic strain and ER stress	UPR, ER-stress, autophagy, degradation programs	Chaperones; phosphorylation and protein-folding pathways	Secondary energy and redox stress	Mainly hepatocytes	Higher maintenance burden and lower stress tolerance (Liu et al., 2022; Puppala et al., 2023)
Inflammatory sensitization and cell–cell crosstalk	Endothelial, macrophage, neutrophil, hepatocyte programs	Cytokines, chemokines, alarmins, adhesion proteins	Injury-associated metabolic shifts	LSECs, macrophages, neutrophils, hepatocytes	Amplified multicellular injury response (Wang et al., 2023; Liu et al., 2024 ; 2026)
Reduced recovery capacity	Regeneration, Hedgehog, adaptive-response programs	ECM, growth-factor, repair and synthesis pathways	Lactate clearance; bile and energy recovery	Hepatocytes, cholangiocytes, stellate cells	Recovery trajectory after initial injury (Maeso-Díaz et al., 2022; Nie et al., 2022; Liu et al., 2025)

The modules are interpretive categories synthesized from the manuscript narrative and curated literature matrix. They organize transcriptomic, proteomic, and metabolomic evidence, but should not be interpreted as validated biomarkers, composite scores, or clinically deployable predictors.

Abbreviations: 13-HODE, 13-hydroxyoctadecadienoic acid; ECM, extracellular matrix; ER, endoplasmic reticulum; FMN, flavin mononucleotide; LSEC, liver sinusoidal endothelial cell; OXPHOS, oxidative phosphorylation; UPR, unfolded protein response; FA, fatty acid.

We use these modules to organize evidence across pathways and contexts; they do not constitute a molecular score or clinical decision tool. Mitochondrial vulnerability links the five modules but is neither a separate module nor a validated measure of biological age.

### 2.4 Interpreting evidence across layers and contexts

Evidence is weighted here by biological coherence, replication across layers or systems, and translational plausibility. Cross-layer and cross-species agreement strengthens a module, whereas a finding from one model or matrix remains exploratory even when mechanistically plausible (Williams et al., 2022; Yang et al., 2024).

Mechanistic, cross-species, and translational studies address causality, conservation, and clinical measurability, respectively. Evidence in one category does not establish readiness in another, particularly when rodent mechanisms have not been linked to human tissue, perfusate, bile, or outcomes (Allaire and Gilgenkrantz, 2020; Hautz et al., 2024).

Machine perfusion links tissue biology with serial fluid readouts under controlled stress and is therefore most useful for translational validation. Procurement, ischemia, donor factors, and protocol also influence these measurements. Current omics signals are best treated as candidate mechanisms and readouts until they are tested prospectively against clinical outcomes (Mergental et al., 2020; Melandro et al., 2022; Eden et al., 2025).

### 2.5 Data integration across omics layers: analytical strategies and bottlenecks

End-to-end integration remains difficult because transcriptomic, proteomic, and metabolomic data differ in scale, missingness, and noise. Most liver-aging studies therefore integrate at the level of pathway concordance rather than through a single fused model (Argelaguet et al., 2020; Williams et al., 2022; Tang et al., 2023).

Joint dimensionality reduction, network analysis, and supervised feature prioritization may be useful when cohorts are sufficiently large and matched. In current liver-aging datasets, module-level integration is generally more robust and interpretable than a compressed multi-omics score (Argelaguet et al., 2020; Subramanian et al., 2020).

## 3 Cross-species evidence for liver aging across omics layers

Evidence for liver aging now spans rodents, non-human primates, and humans, but these sources do not contribute equally to the field. Rodent studies still provide most of the mechanistic depth and omics integration, non-human primate data strengthen confidence in biological conservation, and human studies remain essential for clinical relevance despite greater heterogeneity and confounding. Synthesizing these lines of evidence reveals greater convergence at the level of pathways and biological modules than at the level of individual readouts. Representative studies that anchor this framework are summarized in Table 3.

**TABLE 3:** Representative studies informing major biological modules of liver aging.

Study	Omics layer(s)	Species/model	Biological setting	Key contribution/module relevance
Williams et al. (2022)	Transcriptomics, proteomics, metabolomics	BXD mice; young vs. aged; standard/high-fat diet	Baseline aging; diet–age interaction	Age-, genotype-, and diet-dependent hepatic aging; supports lipid and inflammatory modules
Wang et al. (2024a)	Transcriptomics, metabolomics	Mouse liver and hepatocyte model	Baseline aging; mechanistic integration	FGF21-regulated lipid metabolism, oxidative stress, and recovery; supports lipid and redox modules
Nikopoulou et al. (2023); Sinha et al. (2026)	Spatial and single-cell multi-omics	Mouse and human liver	Baseline aging; spatial heterogeneity	Spatially heterogeneous hepatic aging; supports zonation-aware metabolic interpretation
Schreiter et al. (2021)	Transcriptomics	Human liver tissue	Baseline human aging	Human transcriptomic anchor for baseline aging; supports cautious interpretation of functional gene changes
Puppala et al. (2023); Yang et al. (2024)	Multi-omics; single-nucleus transcriptomics	Non-human primate liver	Cross-species conservation	Conserved primate ER-stress, protein-degradation, and inflammatory programs; supports proteostatic and inflammatory modules
Duan et al. (2023)	Metabolomics with mechanistic validation	Mouse and *in vitro* cell model	Mechanistic aging biology	13-HODE-mediated catalase inhibition and steatosis; links lipid stress to reduced oxidative reserve
Liu et al. (2022); Mohallem et al. (2025)	Proteomics; phosphoproteomics	Mouse liver; young vs. aged	Baseline aging; signaling regulation	Age-related protein-abundance and phosphorylation remodeling; supports proteostatic and signaling modules
Lv et al. (2023)	Metabolic phenotyping and intervention	Aged mouse model	Mechanistic metabolic vulnerability	FABP4 as an aging-sensitive metabolic node; supports lipid stress and metabolic remodeling
Ding et al. (2025)	Proteomics	Human multi-tissue atlas including liver	Human aging context	Human protein-aging context including liver signals; supportive but not liver-specific validation
Liu et al. (2026)	Single-cell transcriptomics	Aged mouse liver I/R model	Stress-exposed inflammatory vulnerability	LSEC–hepatocyte IL-1α–IL1R1 signaling under I/R stress; supports inflammatory and cell–cell crosstalk modules
Han et al. (2021)	Metabolomics	Rat young donor-to-old recipient OLT model	Transplant stress model	Transplant stress-associated lipid and metabolic changes; supports metabolic modules under graft stress
Schlegel et al. (2020); Bhat et al. (2021); Hautz et al. (2024)	Perfusion readouts, metabolomics, transcriptomics where available	Human liver grafts during HOPE/NMP	Machine perfusion validation window	Stress-exposed HOPE/NMP readouts; supports candidate-module validation, not baseline aging definition

Representative studies were selected to anchor the narrative framework across baseline aging, cross-species evidence, mechanistic modules, and stress-exposed translational settings. The table is selective rather than exhaustive. Listed findings support biological interpretation and should not be interpreted as validated clinical biomarkers or decision-ready predictors.

Abbreviations: 13-HODE, 13-hydroxyoctadecadienoic acid; BXD, BXD, recombinant inbred mouse strains; HOPE, hypothermic oxygenated perfusion; I/R, ischemia–reperfusion; LSEC, liver sinusoidal endothelial cell; NMP, normothermic machine perfusion; OLT, orthotopic liver transplantation.

### 3.1 Rodent evidence

Rodent studies provide the clearest evidence that liver aging involves coordinated alterations in transcriptional programs, protein regulation, phosphorylation-state control, and metabolite composition. Large-scale mouse multi-omics datasets show that age-related hepatic phenotypes are shaped not only by age itself, but also by genotype and diet. This observation challenges the assumption that liver aging can be reduced to a single conserved molecular signature. Instead, these studies support a pathway-level view in which age-associated biology emerges from interacting networks of metabolic regulation, stress adaptation, and context-dependent molecular divergence (Williams et al., 2022). Most rodent aging studies are performed in controlled genetic, dietary, and environmental settings; this improves mechanistic clarity but limits direct transfer to the metabolically heterogeneous donor population encountered in clinical transplantation.

Natural aging models that integrate metabolomics and phosphoproteomics reinforce this interpretation. Rather than identifying one dominant lesion, they indicate that age-related vulnerability is distributed across broad functional systems, including lipid metabolism, immune signaling, stress-associated kinase networks, and protein-state regulation. Differences between genetically diverse mouse cohorts and naturally aged inbred models also caution against treating rodent aging trajectories as universal. This has two important implications. First, liver aging appears to involve coordinated remodeling rather than catastrophic collapse. Second, what is seen depends in part on the omics layer under study: transcriptomics may emphasize pathway tendency, phosphoproteomics may capture signaling execution, and metabolomics may reveal physiological consequence (Liu et al., 2022; Ling et al., 2023; Tang et al., 2023; Mohallem et al., 2025).

Mechanistic work in rodents has helped link these layers to specific aging-related processes. A key transcriptome–metabolome study linked age-related changes in peroxisomal and fatty-acid metabolic programs with FGF21 signaling, suggesting that some liver-aging signals may be informative not only at the tissue level but also in more accessible matrices (Wang et al., 2024a). Studies of oxidized lipid mediators further show that senescence-associated 13-HODE can directly inhibit catalase activity and promote age-related steatosis (Duan et al., 2023), while other work implicates dysregulated Hedgehog signaling in diminished recovery capacity (Maeso-Díaz et al., 2022). Such mechanisms link the aging phenotype to an impaired response after challenge, moving beyond baseline dysfunction alone (Lv et al., 2023).

High-resolution spatial and single-cell studies have clarified the spatial and cellular heterogeneity of hepatic aging. These data indicate that hepatocytes do not age uniformly across the lobule: pericentral hepatocytes preferentially accumulate large lipid droplets, whereas periportal cells show more prominent mitochondrial and epigenetic disturbance. This spatial pattern means that liver aging cannot be reduced to a single parenchymal average and helps explain why small biopsies or bulk assays may miss biologically important local variation (Doshida et al., 2023; Nikopoulou et al., 2023; Tak et al., 2025; Sinha et al., 2026).

Rat studies add a useful systems-level perspective. They often make endocrine and sex-related modifiers easier to detect than mouse models, and naturally aged rat liver has shown clear sex-dependent differences in senescence and cell-cycle pathways. For translational work, especially in donor assessment models, such modifiers should therefore be incorporated explicitly rather than averaged away (Lomas-Soria et al., 2021).

### 3.2 Non-human primate evidence

Although fewer in number, non-human primate studies occupy an important strategic position in this literature. They reduce the likelihood that a candidate signal is merely rodent-specific and help identify biology that is more likely to persist across species. Existing data support at least partial conservation of age-associated changes in inflammatory signaling, lipid metabolism, oxidative stress, and stress-response pathways. In particular, single-nucleus and multi-omics studies in primate liver have strengthened interest in proteostatic strain, endoplasmic reticulum stress, degradation pathways, and hepatocyte-specific pro-senescence programs as shared features of liver aging (Castro et al., 2012; Puppala et al., 2023; Yang et al., 2024).

The principal value of primate data lies in prioritization rather than in direct clinical validation. When a process is observed in both rodents and primates, confidence increases that it reflects a conserved aging-related module rather than a model-specific artifact. Repeated emphasis on ER stress and degradation pathways, for example, suggests that proteostatic burden is unlikely to be merely a feature of short-lived rodent aging and is better understood as a broader component of liver-aging biology (Castro et al., 2012; Puppala et al., 2023; Yang et al., 2024; Ding et al., 2025; Mohallem et al., 2025).

At the same time, these datasets should be interpreted with restraint. Current primate studies remain limited in size, are often narrow in sex and health status, and do not reproduce the full sequence of procurement, preservation, reperfusion, and implantation that defines clinical transplantation. Some of the most comprehensive primate multi-omics studies to date were also performed in female animals, which limits assessment of sex-dependent aging effects and reduces direct generalizability to heterogeneous donor populations. Their main contribution is therefore biological plausibility: they strengthen mechanistic confidence without, by themselves, resolving questions of clinical translation.

### 3.3 Human evidence

At baseline, human liver aging often appears subtler than preclinical models might suggest. Transcriptomic studies have identified age-related differences in selected functional gene sets, but these signals are frequently entangled with steatosis, fibrosis, metabolic disease, environmental exposure, and medication history. This complicates interpretation because chronological age in clinical cohorts rarely maps cleanly onto biological aging; more often, it reflects a composite of age, metabolic burden, and cumulative exposure history (Bacalini et al., 2019; Durand et al., 2019; Morsiani et al., 2019; Allaire and Gilgenkrantz, 2020; Schreiter et al., 2021).

For this reason, current human evidence remains strongest for transcriptomic change, with selected epigenetic studies providing supporting context, rather than for fully integrated multi-omics characterization. Baseline human liver aging is therefore still defined mainly by transcriptomic and limited complementary evidence, whereas matched human tri-omics datasets remain sparse (Bacalini et al., 2019; Schreiter et al., 2021; Bozukova et al., 2022; Ding et al., 2025; Sinha et al., 2026). Despite these limitations, available studies support the view that baseline liver aging in humans is real but relatively buffered, such that molecular approaches are often required to detect changes that remain invisible to routine clinical descriptors. This has motivated perfusion-based trajectories and other outcome-linked anchors for translational interpretation (Bhat et al., 2021; Hautz et al., 2024; Eden et al., 2025).

Human proteomic and metabolomic evidence is constrained by similar limitations. Matched baseline multi-omics cohorts remain scarce, and many clinically accessible datasets are heterogeneous or enriched for metabolically abnormal states. Broader human aging atlases are helpful in showing that protein-level aging biology and transcriptome–proteome divergence are observed across tissues, but they do not substitute for liver-specific baseline studies. Given these constraints, most omics-derived signals in human liver are still better regarded as candidate biology than as established biomarkers (Bacalini et al., 2019; Schreiter et al., 2021; Melandro et al., 2022; Ding et al., 2025).

These limitations help explain why stress-exposed clinical settings have become so prominent. In transplantation and machine perfusion, vulnerabilities that are difficult to resolve at baseline may become more interpretable under controlled stress exposure (Schlegel et al., 2020; Bhat et al., 2021; Han et al., 2021; Hautz et al., 2024). Importantly, these contexts should be treated as applications of aging biology rather than substitutes for baseline aging evidence. Human liver aging cannot be inferred from transplant stress biology alone, and the current human evidence base remains stronger for mechanistic interpretation than for direct clinical classification (Durand et al., 2019; Allaire and Gilgenkrantz, 2020; Melandro et al., 2022).

### 3.4 What cross-species comparison adds

Cross-species comparison contributes more than breadth; it changes the strength of the claims that can reasonably be made. A pathway observed in a single mouse strain under one dietary condition may be intriguing, but evidence across genetically diverse mice, non-human primates, and selected human datasets makes it less likely that a finding reflects model-specific noise (Schreiter et al., 2021; Williams et al., 2022; Puppala et al., 2023; Yang et al., 2024). That does not mean one should expect identical marker sets across species. More realistically, convergence is seen at the level of process: different studies may nominate different transcripts, proteins, or metabolites while still pointing to the same broader themes, including lipid stress, redox decline, proteostatic burden, inflammatory sensitization, and impaired recovery capacity (Han et al., 2021; Williams et al., 2022; Ding et al., 2025). This is where the module framework becomes useful, because it preserves biological continuity even when marker-level overlap is limited.

Cross-species comparison also clarifies the limits of inference. Rodent studies offer mechanistic resolution under conditions of experimental control that human cohorts cannot approach. Human studies, by contrast, define relevance, heterogeneity, and the practical boundaries of interpretation. Non-human primate work occupies an intermediate position. Cross-species comparison therefore supports a cautious conclusion: the most stable features of liver aging are process-level changes, especially those that become functionally visible under stress.

Evidence maturity differs across modules. Lipid/metabolic remodeling and redox vulnerability have the broadest rodent mechanistic support and limited primate or human anchors; proteostatic strain is supported mainly by rodent and primate omics, whereas inflammatory sensitization and reduced recovery capacity rely heavily on mouse challenge models and hepatocyte-repopulation studies. Translational risk is therefore highest when these tissue findings are treated as clinical biomarkers without validation in human liver, perfusate, or bile. Supplementary Table S1 classifies representative evidence as physiological baseline, challenge-model, or machine-perfusion data.

## 4 Mitochondrial vulnerability modules across omics layers

Age-related alterations in the liver become more coherent when grouped into interconnected vulnerability modules. Across transcriptomic, proteomic, phosphoproteomic, and metabolomic studies, similar patterns emerge despite differences in species, aging models, and sampling contexts (Williams et al., 2022; Puppala et al., 2023; Mohallem et al., 2025). These patterns include lipid and metabolic remodeling, declining oxidative reserve, proteostatic strain, inflammatory sensitization, and reduced recovery capacity (Hunt et al., 2019; Nikopoulou et al., 2023). Mitochondrial dysfunction provides a plausible biological interface through which these five disturbances may reinforce one another.

Organizing the evidence in this way has two advantages. First, it better reflects the biology of liver aging itself, which is distributed across pathways and cell states rather than encoded in one universal marker. Second, it allows evidence from rodents, non-human primates, and humans to be compared without implying that every signal is equally mature, specific, or close to clinical translation. In the discussion below, transplantation and ischemia–reperfusion stress are treated as settings in which these modules may become more visible, rather than as the biological definition of the modules themselves. The major biological modules and their cross-omics support are summarized in Figure 1, which highlights shared patterns of dysfunction across multiple omics layers rather than a single marker. The cross-omics evidence and representative readouts supporting each module are summarized in Table 2.

### 4.1 Lipid stress and metabolic remodeling

Lipid stress is one of the clearest intersections between hepatic aging and mitochondrial vulnerability. Age-associated changes in fatty-acid handling, lipid-droplet distribution, peroxisomal programs, and FGF21/FABP4 signaling can narrow metabolic flexibility even when baseline function is preserved (Williams et al., 2022; Nikopoulou et al., 2023; Lv et al., 2023; Wang et al., 2024a). A mechanistic hepatic model provides one link to the next module: chronic enrichment of ER-mitochondria contacts increased IP3R1-dependent mitochondrial Ca2+ loading, reduced oxidative capacity, and intensified oxidative and ER stress (Arruda et al., 2014). Because that study modeled obesity rather than physiological aging, the mechanism is plausible but still requires direct testing in aged liver. Related MASLD work also implicates long non-coding RNAs in lipid synthesis, fatty-acid β-oxidation, oxidative stress, and metabolic inflammation, although these findings are not aging-specific (Wade et al., 2024).

### 4.2 Oxidative reserve and redox vulnerability

Reduced oxidative reserve is better represented by a panel of functional readouts than by 13-HODE alone. Recurring signals in aged rodent and primate liver include an altered GSH/GSSG balance, increased ROS/RNS and malondialdehyde, mtDNA damage or deletions, and changes in catalase, superoxide dismutase, antioxidant proteins, and respiratory-chain components (Castro et al., 2012; Niemann et al., 2017; Bárcena et al., 2021; Huang et al., 2021). In aged mice, 13-HODE directly inhibited catalase and promoted steatosis, providing a specific lipid-redox mechanism (Duan et al., 2023). These measures are candidate readouts rather than interchangeable or clinically validated biomarkers; their meaning depends on matrix, timing, and stress context.

### 4.3 Proteostasis and ER stress

Proteostatic strain includes ER stress, unfolded-protein responses, impaired degradation, and the energetic cost of maintaining protein quality. Rodent phosphoproteomic data and primate omics studies converge on altered chaperone, degradation, and stress-response pathways, although direct human liver validation remains limited (Liu et al., 2022; Puppala et al., 2023; Yang et al., 2024; Mohallem et al., 2025).

This module is mechanistically linked to lipid and redox stress. Excess mitochondrial Ca2+ and ROS can increase ER protein-folding demand, whereas ATP limitation can weaken chaperone and degradation capacity. Proteostatic strain may therefore be a downstream amplifier of metabolic overload rather than an isolated feature (Arruda et al., 2014). Disease-focused evidence further links bile acid signaling with autophagy, lysosomal function, redox homeostasis, and ER stress, adding context for crosstalk among the metabolic, redox, and proteostatic modules (Li et al., 2024).

### 4.4 Inflammatory sensitization and cell–cell crosstalk

Aging also sensitizes the hepatic microenvironment. Single-cell studies identify altered endothelial, macrophage, neutrophil, and hepatocyte programs, while challenge models show that IL-1α–IL1R1 signaling and Cxcl2-positive macrophage recruitment can amplify ischemia–reperfusion injury in aged liver (Wang et al., 2023; Liu et al., 2024; Liu et al., 2026). This module is therefore best defined by coordinated cell–cell signaling rather than by a single circulating cytokine. This multicellular view is also consistent with disease-focused evidence that cytokine- and chemokine-mediated crosstalk among hepatic parenchymal and non-parenchymal cells amplifies liver injury, although these data are not aging-specific (Gao et al., 2024).

### 4.5 Reduced recovery capacity under stress

Recovery capacity describes the ability to restore metabolic, synthetic, and structural homeostasis after injury. Aged-liver studies implicate Hedgehog dysregulation, reduced hepatocyte plasticity, and extracellular-matrix remodeling in impaired regeneration (Maeso-Díaz et al., 2022; Nie et al., 2022; Liu et al., 2025). This endpoint is distinct from initial injury burden: two grafts may sustain similar early stress yet diverge in lactate clearance, bile production, synthetic recovery, or survival.

### 4.6 Mitochondrial convergence across the five biological modules

The five modules are mechanistically connected. Lipid overload can increase mitochondrial Ca2+ and ROS, which promotes ER stress and inflammatory signaling; proteostatic and inflammatory burden then consumes energy and limits repair, reducing recovery capacity. Feedback in the opposite direction is also likely, because failed proteostasis and inflammation further impair mitochondrial quality control and metabolic flexibility (Arruda et al., 2014; Hunt et al., 2019; Maeso-Díaz et al., 2022).

This network generates testable predictions. A liver with combined lipid stress and low redox reserve should show a larger stress-induced rise in mitochondrial, ER-stress, and inflammatory readouts and a slower recovery trajectory than a liver with only one abnormal module. Longitudinal, matrix-matched studies are needed to test whether such module combinations predict outcomes better than donor age or any single analyte.

### 4.7 Translational use and limitations of the module framework

In practice, the module framework helps select the appropriate matrix, sampling interval, and validation endpoint. Tissue captures pathway state, serial perfusate captures stress and recovery kinetics, bile captures cholangiocyte function, and post-transplant outcomes provide clinical validation. It can guide compact, hypothesis-driven panels, but it does not yet support a clinical score.

## 5 Transplantation and machine perfusion as stress tests of mitochondrial vulnerability

Transplantation is one of the clearest clinical settings in which liver aging becomes functionally visible. From a mitochondrial perspective, procurement, cold preservation, reperfusion, and metabolic reactivation impose rapid changes in oxygen availability and energetic demand. Transplantation therefore provides a clinically relevant stress test of mitochondrial reserve, although it does not directly measure mitochondrial aging. Age-associated alterations that may appear modest at baseline, including reduced redox reserve, inflammatory sensitization, and impaired recovery capacity, can be amplified during organ procurement and preservation, extending through the phases of implantation and reperfusion. In this sense, transplantation does not redefine liver aging, but instead exposes consequences that are often difficult to appreciate under resting conditions.

Machine perfusion is useful in this context because it creates a controlled *ex vivo* interval during which tissue and fluid can be sampled under stress (Schlegel et al., 2020; Bhat et al., 2021; Hautz et al., 2024; Eden et al., 2025). Biopsies may help characterize baseline programs, whereas serial perfusate or bile measurements can capture short-term injury and recovery trajectories. Its value in this review is therefore selective rather than absolute: machine perfusion is treated not as a definitive assessment tool, but as a setting in which biologically related modules can be challenged and compared with more accessible readouts (Mergental et al., 2020; Melandro et al., 2022).

### 5.1 Why transplantation reveals aging-related differences under stress

The transplant setting can make differences in reserve, inflammatory control, and recovery more apparent than they are at baseline. This is one reason why some of the clearest human signals in the field emerge under ischemia–reperfusion stress rather than from static donor tissue alone. The IL-1α–IL1R1 axis is one example of an age-amplified injury mechanism that would have been difficult to infer from baseline histology or routine fluid markers (Liu et al., 2026). More broadly, transplantation shows that clinically relevant aspects of liver aging often become interpretable only when reserve is challenged.

In practice, grafts with similar donor age and broadly comparable biopsy steatosis may still diverge in early post-implant function. That discrepancy reflects stress vulnerability rather than baseline appearance (Durand et al., 2019; Kanneganti et al., 2023; Shirini et al., 2026). Transplantation provides a useful setting to test whether a proposed biological module captures injury amplification, impaired reserve, or delayed recovery. This context also supports calibration against clearly defined early clinical endpoints (Melandro et al., 2022).

### 5.2 Machine perfusion as a validation window

Randomized trials have established the clinical utility of hypothermic oxygenated perfusion (HOPE) and normothermic machine perfusion (NMP) (Nasralla et al., 2018; Van Rijn et al., 2021). HOPE reduced early allograft dysfunction and symptomatic non-anastomotic biliary strictures in donation after circulatory death (DCD) liver transplantation (Van Rijn et al., 2021), whereas NMP reduced biochemical graft injury and organ discard without compromising graft survival (Nasralla et al., 2018). Within the framework shown in Figure 1, machine perfusion provides a validation setting for stress-exposed biology, even if it cannot directly measure baseline aging. Recent studies support this potential, showing that normothermic machine perfusion can converge the transcriptomic profiles of high- and low-risk grafts, reducing inflammatory signals and promoting repair pathways (Keslar et al., 2025). Therapeutic interventions during perfusion, such as mild hyperthermia, have been shown to improve graft physiology by promoting lipid export and stress response pathways, positioning machine perfusion not just as a preservation tool but as a therapeutic platform (Thorne et al., 2025). This *ex vivo* setting can link tissue-level biology with measurable fluid signals and transplant-related outcomes (Hautz et al., 2024; Eden et al., 2025).

Human perfusion studies now provide several concrete examples of stress-exposed readouts. During HOPE, early perfusate flavin mononucleotide (FMN) was higher in grafts that developed early allograft dysfunction (EAD); in a 50-graft validation cohort, the 5-min value yielded an area under the curve of 0.744 for EAD and 0.890 for 1-year mortality (Dingfelder et al., 2025). International data further support the use of FMN trajectories as a measure of mitochondrial injury during HOPE (Eden et al., 2025). During NMP, serial perfusate AST, ALT, and LDH, together with von Willebrand factor and platelet-related changes, were associated with EAD in 45 transplanted grafts (Weissenbacher et al., 2022). These studies support trajectory-based assessment, but they do not establish aging specificity.

### 5.3 Conceptual links between biological modules and transplant-relevant outcomes

Table 4 maps each biological module to a clinically relevant phenotype and now includes representative human perfusion readouts. The examples are intended to make the framework operational, not to imply that the modules have already been converted into a validated composite score.

**TABLE 4:** Hypothesis-generating links between liver-aging modules and transplant stress outcomes.

Module	Clinical phenotype	Representative human readout	Evidence boundary	Next validation step
Lipid/metabolic remodeling	EAD; unstable metabolic recovery	NMP metabolite and enzyme trajectories	Mechanistic support; limited age-specific outcome linkage (Bhat et al., 2021; Weissenbacher et al., 2022)	Test added value beyond donor age, steatosis, and ischemia
Reduced oxidative reserve	Severe I/R injury; EAD; mortality	HOPE FMN trajectory; lactate clearance	FMN associated with EAD and survival, but not aging-specific (Dingfelder et al., 2025; Eden et al., 2025)	Link fluid kinetics to tissue redox programs
Proteostatic strain	Delayed synthetic recovery	Candidate UPR/chaperone proteins in tissue or perfusate	Cross-species omics; no validated clinical panel	Prospective tissue-fluid pairing
Inflammatory sensitization	EAD; biliary injury; graft loss	NMP IL-6, TNF-α, IL-10	Small cohort associations with bilirubin, INR, ICU stay, or graft loss (Pezzati et al., 2025)	External validation with standardized endpoints
Reduced recovery capacity	Delayed recovery; cholangiopathy	Bile pH, bicarbonate, glucose; enzyme/lactate trajectories	Associated with bile-duct injury or outcomes in small series (Watson et al., 2018; Matton et al., 2019)	Serial validation against EAD, NAS, and graft survival

The links shown here are conceptual and hypothesis-generating rather than outcome-validated. Clinical outcomes likely reflect overlapping contributions from multiple modules. Future validation should adjust for key confounders, including donor age, steatosis, donor metabolic status, DCD, status, ischemia time, and perfusion protocol.

Abbreviations: ATP, adenosine triphosphate; DCD, donation after circulatory death; EAD, early allograft dysfunction; ER, endoplasmic reticulum; FAO, fatty-acid oxidation; FMN, flavin mononucleotide; HOPE, hypothermic oxygenated perfusion; I/R, ischemia–reperfusion; IC, ischemic cholangiopathy; LSEC, liver sinusoidal endothelial cell; NAS, non-anastomotic stricture; NMP, normothermic machine perfusion; ROS, reactive oxygen species; UPR, unfolded protein response.

The purpose of this mapping is not to assign each module to a single endpoint, because in practice transplant outcomes are likely to reflect overlapping contributions from several modules at once. Rather, it is to clarify translational logic by indicating which dimensions of vulnerability are most plausibly connected to which categories of outcome. Metabolic fitness is likely to align with redox and mitochondrial injury signals that worsen reperfusion damage (Schlegel et al., 2020; Bhat et al., 2021; Eden et al., 2025). Inflammatory stress is more likely to correspond to cytokine release and niche-level crosstalk that amplify ischemia–reperfusion injury (Liu et al., 2024; 2026). Recovery capacity, in turn, is most relevant to repair programs and post-implant resilience (Maeso-Díaz et al., 2022; Nie et al., 2022; Hautz et al., 2024). For this reason, future studies should predefine endpoints such as EAD, biliary injury including IC or symptomatic non-anastomotic strictures (NAS), and graft loss, and then test whether module-based readouts provide information beyond age and steatosis alone (Melandro et al., 2022).

### 5.4 What is clinically anchored and what remains exploratory

Inflammatory and biliary measurements provide additional clinical anchors. In a 30-graft NMP series, perfusate IL-6, TNF-α, and IL-10 were associated with postoperative bilirubin or INR peaks, intensive-care stay, or graft loss (Pezzati et al., 2025). Bile pH, bicarbonate, glucose, and the bile-to-perfusate glucose ratio have been associated with bile-duct injury and post-transplant cholangiopathy risk during NMP (Watson et al., 2018; Matton et al., 2019). These small cohorts require external validation, but they show how inflammatory and recovery modules can be linked to measurable clinical endpoints.

No published study has validated a combined FMN-cytokine panel across HOPE and NMP. FMN, enzyme trajectories, cytokines, and biliary chemistry were measured in different protocols and cohorts; they should therefore not be merged into a decision rule without prospective, matrix-matched validation. Their current value is to define a testable pipeline in which tissue modules are linked to serial fluid readouts and then to standardized EAD, biliary, and graft-survival endpoints.

## 6 Discussion: mechanistic implications and potential intervention relevance

Omics studies of the aging liver converge on three mechanistically relevant axes: lipid handling, oxidative reserve, and recovery signaling. These pathways provide testable hypotheses for experimental work, although evidence for therapeutic translation remains limited.

### 6.1 Candidate mechanistic targets for intervention: from metabolism to recovery

FGF21 and FABP4 connect transcriptional regulation with protein and metabolite changes, making them useful targets for metabolism-focused experiments (Lv et al., 2023; Wang et al., 2024a). The 13-HODE-catalase axis similarly points to impaired antioxidant buffering as a source of vulnerability (Bárcena et al., 2021; Duan et al., 2023). Hedgehog signaling and hepatocyte regenerative reserve shift attention from initial injury to the capacity for recovery (Maeso-Díaz et al., 2022; Nie et al., 2022). These pathways now require testing in stress-exposed models and machine perfusion before intervention claims can be made.

### 6.2 Translational cautions and the path toward clinical testing

Evidence for therapeutic translation remains limited. For now, machine perfusion is most useful for testing specific mechanistic hypotheses under controlled stress. Any intervention claim will require direct evidence of benefit against clinically meaningful outcomes.

## 7 Limitations and future directions

The liver-aging literature has expanded, but its evidentiary depth remains uneven across species, study designs, and omics layers. Mechanistic resolution still comes predominantly from rodent models, non-human primate studies mainly strengthen the case for biological conservation, and human evidence, although growing, remains constrained by heterogeneity and the scarcity of integrated baseline multi-omics datasets (Schreiter et al., 2021; Williams et al., 2022; Puppala et al., 2023; Yang et al., 2024; Ding et al., 2025). In addition, observed signals are strongly influenced by sex, strain, diet, sampling matrix, and stress model, while in clinical cohorts aging is often difficult to disentangle from steatosis, metabolic disease, chronic inflammation, and treatment exposure.

Progress will depend on aligning biological context, omics layer, and intended inference. Paired tissue-fluid datasets, explicit separation of baseline aging from stress responses, and validation in well-phenotyped human cohorts are the immediate priorities. The near-term goal is a clearer link between tissue biology, measurable readouts, and clinical outcomes, not a finished scoring system.

### 7.1 Current evidence gaps

Several major gaps continue to limit the field. In clinical practice, truly healthy aged liver tissue is difficult to obtain for ethical and logistical reasons, so human datasets often reflect mixtures of physiological aging, metabolic disease, steatosis, fibrosis, inflammation, and treatment exposure. Baseline human multi-omics data remain scarce, the separation of aging from comorbidity is often incomplete, and the relationship between tissue-level programs and accessible fluid-based signals is still poorly defined (Bacalini et al., 2019; Schreiter et al., 2021; Hautz et al., 2024; Ding et al., 2025). A further problem is the mismatch between mechanistic and translational maturity: some pathways are biologically compelling but clinically remote, whereas some clinically useful indicators remain only weakly explained at the mechanistic level.

These limitations are particularly relevant for clinical translation. Human datasets rarely provide paired tissue and perfusate measurements at fixed time points, which restricts transferability of candidate markers across settings (Bhat et al., 2021; Hautz et al., 2024; Eden et al., 2025). Aging-related signals are also easily confounded by steatosis and metabolic disease history. This clinical overlap necessitates careful adjustment and highlights the value of designs that reduce systemic confounding, such as machine perfusion. In addition, perfusion protocols and readouts vary across centers, complicating pooling and threshold learning, while outcome definitions and follow-up windows, especially for biliary endpoints, remain insufficiently harmonized for robust external validation.

### 7.2 Priorities for future studies

A practical next step is to develop large, paired tissue-fluid biobanks that link baseline donor biopsies with serial perfusate and post-transplant samples. Sampling windows should distinguish immediate procurement injury from sustained functional recovery. Circadian timing and other pre-analytical factors also require attention, particularly for metabolomic readouts (Buijink et al., 2024). Single-cell and spatial omics can strengthen biopsy interpretation and help identify where targeted assays should sample (Nikopoulou et al., 2023; Vandereyken et al., 2023; Lin et al., 2024; Sinha et al., 2026). A recent synthesis of acute liver injury and regeneration shows how single-cell and spatial transcriptomics can resolve hepatocyte, stellate-cell, endothelial, and macrophage states across injury-repair trajectories (Wen and Ju, 2025).

Several methodological issues also warrant greater attention. One is zonation: the liver is spatially organized, and aging-related changes in metabolism, mitochondrial status, and chromatin state are not evenly distributed across the lobule, so small biopsy specimens may carry substantial spatial variance (Nikopoulou et al., 2023; Sinha et al., 2026). A second is timing. In perfusion studies, early readouts may primarily reflect procurement injury and ischemic burden. By contrast, later measurements may better capture true recovery behavior. Without an explicit sampling-timing framework, these distinct phases risk collapsing into a single, poorly interpretable signal.

A third issue concerns the relationship between aging and disease. Many human liver samples are not examples of physiological aging alone, but instead reflect varying combinations of metabolic burden, fibrosis, steatosis, and inflammation. That does not diminish their relevance, because in clinical practice such overlap is part of the problem; however, it does mean that liver-aging studies should avoid treating all older human samples as interchangeable with healthy aging. More explicit characterization of this overlap would improve both biological interpretation and translational relevance.

A fourth issue is assay feasibility. Transcriptomic, proteomic, and metabolomic signals do not share the same clinical applicability. Some can fit within a perfusion workflow and some cannot; some remain robust across matrices, whereas others are sensitive to pre-analytical handling. ATP and other energy-related metabolites can be sensitive to sampling and processing conditions, while metabolomic readouts may also vary with ischemic exposure and circadian timing (Bhat et al., 2021; Buijink et al., 2024). By contrast, more stable secreted proteins in perfusate may be easier to standardize. In practical terms, useful assays must return results within hours, add value beyond age and steatosis, and be tested within time-compatible machine perfusion workflows that link tissue-defined biology with serial fluid readouts before implantation (Bhat et al., 2021; Hautz et al., 2024; Eden et al., 2025).

Future studies should combine multi-omics profiling with functional measurements of mitochondrial state, including respiration, ATP production, redox ratios, mitochondrial DNA damage, and dynamic metabolite trajectories (Schlegel et al., 2020; Bhat et al., 2021; Nikopoulou et al., 2023; Eden et al., 2025). Paired tissue, perfusate, and bile sampling may help determine whether omics-derived mitochondrial signals reflect baseline aging, procurement injury, or the capacity for metabolic recovery (Bhat et al., 2021; Hautz et al., 2024; Eden et al., 2025).

## 8 Conclusion

Liver aging is best understood not as a uniform decline with chronological age, but as a set of interacting vulnerabilities involving metabolic remodeling, reduced redox reserve, proteostatic strain, inflammatory sensitization, and impaired recovery after stress. Mitochondrial vulnerability may provide a unifying biological interface across these modules without implying that liver aging can be reduced to a single mitochondrial defect. This framework helps explain why aging livers with similar chronological age or histological appearance may behave differently when exposed to metabolic, toxic, or transplantation-related stress.

The current evidence remains uneven. Rodent and non-human primate studies provide much of the mechanistic support, whereas integrated baseline multi-omics data from human liver are still limited. Transplantation and machine perfusion are therefore useful stress-exposed settings for testing candidate mechanisms and measurable readouts, but they should not be treated as substitutes for baseline aging biology or as validated clinical decision tools.

A module-based interpretation organizes existing omics findings and can guide future studies toward more focused graft assessment, risk-stratification hypotheses, and intervention hypotheses.

## References

[B1] Allaire M. Gilgenkrantz H. (2020). The aged liver: beyond cellular senescence. Clin. Res. Hepatology Gastroenterology 44, 6–11. 10.1016/j.clinre.2019.07.011 31427197

[B2] Argelaguet R. Arnol D. Bredikhin D. Deloro Y. Velten B. Marioni J. C. (2020). MOFA+: a statistical framework for comprehensive integration of multi-modal single-cell data. Genome Biol. 21, 111. 10.1186/s13059-020-02015-1 32393329 PMC7212577

[B3] Arruda A. P. Pers B. M. Parlakgül G. Güney E. Inouye K. Hotamisligil G. S. (2014). Chronic enrichment of hepatic endoplasmic reticulum-mitochondria contact leads to mitochondrial dysfunction in obesity. Nat. Med. 20, 1427–1435. 10.1038/nm.3735 25419710 PMC4412031

[B4] Bacalini M. G. Franceschi C. Gentilini D. Ravaioli F. Zhou X. Remondini D. (2019). Molecular aging of human liver: an epigenetic/transcriptomic signature. Journals Gerontology Ser. A 74, 1–8. 10.1093/gerona/gly048 29554203

[B5] BárcenaB. SalamancaA. PintadoC. MazuecosL. VillarM. MoltóE. (2021). Aging induces hepatic oxidative stress and nuclear proteomic remodeling in liver from wistar rats. Antioxidants 10, 1535. 10.3390/antiox10101535 PMC853312234679670

[B6] Bhat M. Linares-Cervantes I. Pasini E. Patel P. Goto T. Noguchi Y. (2021). Normothermic *Ex Situ* liver perfusion enhances mitochondrial function of DCD grafts as evidenced by high-throughput metabolomics. Transplantation 105, 1530–1538. 10.1097/TP.0000000000003476 33031224

[B7] Bozukova M. Nikopoulou C. Kleinenkuhnen N. Grbavac D. Goetsch K. Tessarz P. (2022). Aging is associated with increased chromatin accessibility and reduced polymerase pausing in liver. Mol. Syst. Biol. 18, MSB202211002. 10.15252/msb.202211002 PMC9459415 36082605

[B8] Buijink M. R. Van Weeghel M. Harms A. Murli D. S. Meijer J. H. Hankemeier T. (2024). Loss of temporal coherence in the circadian metabolome across multiple tissues during ageing in mice. Eur J Neurosci. 60, 3843–3857. 10.1111/ejn.16428 38802069

[B9] Castro M. del R. Suarez E. Kraiselburd E. Isidro A. Paz J. Ferder L. (2012). Aging increases mitochondrial DNA damage and oxidative stress in liver of rhesus monkeys. Exp. Gerontol. 47, 29–37. 10.1016/j.exger.2011.10.002 22027539 PMC3770191

[B10] Ding Y. Zuo Y. Zhang B. Fan Y. Xu G. Cheng Z. (2025). Comprehensive human proteome profiles across a 50-year lifespan reveal aging trajectories and signatures. Cell 188, 5763–5784.e26. 10.1016/j.cell.2025.06.047 40713952

[B11] DingfelderJ. KollmannD. RauterL. PereyraD. KacarS. WeijlerA. M. (2025). Validation of mitochondrial FMN as a predictor for early allograft dysfunction and patient survival measured during hypothermic oxygenated perfusion. Liver Transpl. 31, 476–488. 10.1097/LVT.0000000000000512 PMC1189582539787526

[B12] DoshidaY. HashimotoS. IwabuchiS. TakinoY. IshiwataT. AigakiT. (2023). Single-cell RNA sequencing to detect age-associated genes that identify senescent cells in the liver of aged mice. Sci. Rep. 13, 14186. 10.1038/s41598-023-41352-6 PMC1046852637648885

[B13] DuanJ. DongW. WangG. XiuW. PuG. XuJ. (2023). Senescence-associated 13-HODE production promotes age-related liver steatosis by directly inhibiting catalase activity. Nat. Commun. 14, 8151. 10.1038/s41467-023-44026-z PMC1071042238071367

[B14] Durand F. Levitsky J. Cauchy F. Gilgenkrantz H. Soubrane O. Francoz C. (2019). Age and liver transplantation. J. Hepatology 70, 745–758. 10.1016/j.jhep.2018.12.009 30576701

[B15] Eden J. Thorne A. M. Bodewes S. B. Patrono D. Roggio D. Breuer E. (2025). Assessment of liver graft quality during hypothermic oxygenated perfusion: the first international validation study. J. Hepatology 82, 523–534. 10.1016/j.jhep.2024.08.030 PMC11830552 39251091

[B16] GaoH. JiangY. ZengG. HudaN. ThoudamT. YangZ. (2024). Cell-to-cell and organ-to-organ crosstalk in the pathogenesis of alcohol-associated liver disease. eGastroenterology 2, e100104. 10.1136/egastro-2024-100104 PMC1167400039735421

[B17] HanQ. LiH. JiaM. WangL. ZhaoY. ZhangM. (2021). Age-related changes in metabolites in young donor livers and old recipient sera after liver transplantation from young to old rats. Aging Cell 20, e13425. 10.1111/acel.13425 PMC828223934157207

[B18] HautzT. HacklH. GottschlingH. GronauerR. HofmannJ. SalcherS. (2024). Transcriptomic signatures during normothermic liver machine perfusion correspond with graft quality and predict the early graft function. eBioMedicine 108, 105330. 10.1016/j.ebiom.2024.105330 PMC1142613439299005

[B19] HuangY. L. ShenZ. Q. HuangC. H. LinC. H. TsaiT. F. (2021). Cisd2 slows Down liver aging and attenuates age-related metabolic dysfunction in Male mice. Aging Cell 20, e13523. 10.1111/acel.13523 PMC867279234811857

[B20] Hunt N. J. Kang S. W. (S.) Lockwood G. P. Le Couteur D. G. Cogger V. C. (2019). Hallmarks of aging in the liver. Comput. Struct. Biotechnol. J. 17, 1151–1161. 10.1016/j.csbj.2019.07.021 31462971 PMC6709368

[B21] KannegantiM. OlthoffK. M. BittermannT. (2023). Impact of older donor age on recipient and graft survival after LDLT: the US experience. Transplantation 107, 162–171. 10.1097/TP.0000000000004289 PMC977186736042545

[B22] KeslarK. S. LiuQ. BaldwinW. M. GaudetteB. T. WehrleC. J. SunK. (2025). Normothermic machine perfusion converges transplant induced transcript profiles in low and high risk DBD and DCD livers. Sci. Rep. 15, 34419. 10.1038/s41598-025-17448-6 PMC1249147141039014

[B23] KwongA. J. KimW. R. LakeJ. R. SchladtD. P. HandarovaD. HowellJ. (2025). OPTN/SRTR 2023 annual data report: liver. Am. J. Transplant. 25, S193–S287. 10.1016/j.ajt.2025.01.022 PMC1233419339947804

[B24] LiT. HasanM. N. GuL. (2024). Bile acids regulation of cellular stress responses in liver physiology and diseases. eGastroenterology 2, e100074. 10.1136/egastro-2024-100074 PMC1125707839027418

[B25] Lin P. Yan X. Jing S. Wu Y. Shan Y. Guo W. (2024). Single-cell and spatially resolved transcriptomics for liver biology. Hepatology 80, 698–720. 10.1097/HEP.0000000000000387 37002587

[B26] Ling M. Tang C. Yang X. Yu N. Song Y. Ding W. (2023). Integrated metabolomics and phosphoproteomics reveal the protective role of exosomes from human umbilical cord mesenchymal stem cells in naturally aging mouse livers. Exp. Cell Res. 427, 113566. 10.1016/j.yexcr.2023.113566 37004949

[B27] LiuJ.-F. WuY. YangY.-H. WuS.-F. LiuS. XuP. (2022). Phosphoproteome profiling of mouse liver during normal aging. Proteome Sci. 20, 12. 10.1186/s12953-022-00194-2 PMC935436035932011

[B28] LiuY. XiaoJ. CaiJ. LiR. SuiX. ZhangJ. (2024). Single-cell immune profiling of mouse liver aging reveals Cxcl2+ macrophages recruit neutrophils to aggravate liver injury. Hepatology 79, 589–605. 10.1097/HEP.0000000000000590 PMC1087158837695548

[B29] LiuJ. SongQ. LiC. YanJ. AnN. YinW. (2025). Deciphering age-dependent ECM remodelling in liver: proteomic profiling and its implications for aging and therapeutic targets. Cell Prolif. 58, e70087. 10.1111/cpr.70087 PMC1241464440537154

[B30] LiuY. WangT. ZhangF. LiuX. HuZ. ZhangJ. (2026). Crosstalk between liver sinusoidal endothelial cells and hepatocytes via IL-1α–IL1R1 axis exacerbates ischaemia/reperfusion injury in aged livers. Gut 75, 1548–1564. 10.1136/gutjnl-2025-335964 PMC1342208541062182

[B31] Lomas‐Soria C. Cox L. A. Nathanielsz P. W. Zambrano E. (2021). Sexual dimorphism in liver cell cycle and senescence signalling pathways in young and old rats. J. Physiology 599, 4309–4320. 10.1113/JP281822 34387378

[B32] Lv J. Hu Y. Li L. He Y. Wang J. Guo N. (2023). Targeting FABP4 in elderly mice rejuvenates liver metabolism and ameliorates aging-associated metabolic disorders. Metabolism 142, 155528. 10.1016/j.metabol.2023.155528 36842611

[B33] Ma S. Ji Z. Zhang B. Geng L. Cai Y. Nie C. (2024). Spatial transcriptomic landscape unveils immunoglobin-associated senescence as a hallmark of aging. Cell 187, 7025–7044.e34. 10.1016/j.cell.2024.10.019 39500323

[B34] Maeso‐Díaz R. Dalton G. D. Oh S. Du K. Tang L. Chen T. (2022). Aging reduces liver resiliency by dysregulating hedgehog signaling. Aging Cell 21, e13530. 10.1111/acel.13530 34984806 PMC8844109

[B35] Matton A. P. M. de Vries Y. Burlage L. C. van Rijn R. Fujiyoshi M. de Meijer V. E. (2019). Biliary bicarbonate, pH, and glucose are suitable biomarkers of biliary viability during *ex situ* normothermic machine perfusion of human donor livers. Transplantation 103, 1405–1413. 10.1097/TP.0000000000002500 30395120 PMC6613725

[B36] Melandro F. De Carlis R. Torri F. Lauterio A. De Simone P. De Carlis L. (2022). Viability criteria during liver *Ex-Situ* normothermic and hypothermic perfusion. Medicina 58, 1434. 10.3390/medicina58101434 PMC9608604 36295593

[B37] Mergental H. Laing R. W. Kirkham A. J. Perera M. T. P. R. Boteon Y. L. Attard J. (2020). Transplantation of discarded livers following viability testing with normothermic machine perfusion. Nat. Commun. 11, 2939. 10.1038/s41467-020-16251-3 32546694 PMC7298000

[B38] MohallemR. SchaserA. J. AryalU. K. (2025). Proteomic and phosphoproteomic signatures of aging mouse liver. GeroScience 47, 5205–5224. 10.1007/s11357-025-01601-0 40087212 PMC12181607

[B39] Morsiani C. Bacalini M. G. Santoro A. Garagnani P. Collura S. D’Errico A. (2019). The peculiar aging of human liver: a geroscience perspective within transplant context. Ageing Res. Rev. 51, 24–34. 10.1016/j.arr.2019.02.002 30772626

[B40] Nasralla D. Coussios C. C. Mergental H. Akhtar M. Z. Butler A. J. Ceresa C. D. L. (2018). A randomized trial of normothermic preservation in liver transplantation. Nature 557, 50–56. 10.1038/s41586-018-0047-9 29670285

[B41] Nie Y. Zheng Y. Taniguchi H. (2022). Improving the repopulation capacity of elderly human hepatocytes by decoding aging‐associated hepatocyte plasticity. Hepatology 76, 1030–1045. 10.1002/hep.32443 35243665

[B42] Niemann J. Johne C. Schröder S. Koch F. Ibrahim S. M. Schultz J. (2017). An mtDNA mutation accelerates liver aging by interfering with the ROS response and mitochondrial life cycle. Free Radic. Biol. Med. 102, 174–187. 10.1016/j.freeradbiomed.2016.11.035 27890640

[B43] NikopoulouC. KleinenkuhnenN. ParekhS. SandovalT. ZiegenhainC. SchneiderF. (2023). Spatial and single-cell profiling of the metabolome, transcriptome and epigenome of the aging mouse liver. Nat. Aging 3, 1430–1445. 10.1038/s43587-023-00513-y PMC1064559437946043

[B44] Pezzati D. Torri F. Franzini M. Balzano E. Catalano G. Tincani G. (2025). Association of perfusate cytokine concentrations during liver graft *ex situ* normothermic perfusion to donor type and postoperative outcomes. Liver Transpl. 31, 877–889. 10.1097/LVT.0000000000000583 39927817

[B45] Puppala S. Chan J. Zimmerman K. D. Hamid Z. Ampong I. Huber H. F. (2023). Multi-omics analysis of aging liver reveals changes in endoplasmic stress and degradation pathways in female nonhuman primates. bioRxiv, 2023.08.21.554149. 10.1101/2023.08.21.554149

[B46] Schlegel A. Muller X. Mueller M. Stepanova A. Kron P. De Rougemont O. (2020). Hypothermic oxygenated perfusion protects from mitochondrial injury before liver transplantation. EBioMedicine 60, 103014. 10.1016/j.ebiom.2020.103014 32979838 PMC7519249

[B47] Schreiter T. Gieseler R. K. Vílchez-Vargas R. Jauregui R. Sowa J.-P. Klein-Scory S. (2021). Transcriptome-wide analysis of human liver reveals age-related differences in the expression of select functional gene clusters and evidence for a PPP1R10-Governed ‘aging Cascade. ’ Pharm. 13, 2009. 10.3390/pharmaceutics13122009 PMC8709089 34959291

[B48] Shirini K. Al Ramlawi A. Longchamp A. Bhati C. Meier R. P. H. (2026). Effect of donor–recipient age difference on liver transplant outcomes. Transplant. Direct 12, e1906. 10.1097/TXD.0000000000001906 41694481 PMC12900182

[B49] Sinha S. Ali Q. Zhang T. T-Nguyen D. H. Hanna S. Sethiadi J. (2026). Aging disrupts hepatocyte zonation homeostasis in mice and humans. Hepatology 83, 1143–1157. 10.1097/HEP.0000000000001451 40622856 PMC12645568

[B50] Subramanian I. Verma S. Kumar S. Jere A. Anamika K. (2020). Multi-omics data integration, interpretation, and its application. Bioinform Biol. Insights 14, 1177932219899051. 10.1177/1177932219899051 32076369 PMC7003173

[B51] Tak K. Y. Kim J. Park M. Kim W. Lee S. Park N. (2025). Quasi-spatial single-cell transcriptome based on physical tissue properties defines early aging associated niche in liver. Nat. Aging 5, 929–949. 10.1038/s43587-025-00857-7 40325195

[B52] Tang C. Zhang Z. Sun Y. Ding W. Yang X. Song Y. (2023). Multi-omics reveals aging-related pathway in natural aging mouse liver. Heliyon 9, e21011. 10.1016/j.heliyon.2023.e21011 37920504 PMC10618800

[B53] ThorneA. M. GengY. LantingaV. A. SmitM. KuivenhovenJ. A. PorteR. J. (2025). Therapeutic hyperthermia promotes lipid export and HSP70/90 during machine perfusion of human livers. Physiol. Rep. 13, e70348. 10.14814/phy2.70348 40346031 PMC12064339

[B54] Van Rijn R. Schurink I. J. De Vries Y. Van Den Berg A. P. Cortes Cerisuelo M. Darwish Murad S. (2021). Hypothermic machine perfusion in liver transplantation — a randomized trial. N. Engl. J. Med. 384, 1391–1401. 10.1056/NEJMoa2031532 33626248

[B55] VandereykenK. SifrimA. ThienpontB. VoetT. (2023). Methods and applications for single-cell and spatial multi-omics. Nat. Rev. Genet. 24, 494–515. 10.1038/s41576-023-00580-2 PMC997914436864178

[B56] Wade H. Pan K. Zhang B. Zheng W. Su Q. (2024). Mechanistic role of long non-coding RNAs in the pathogenesis of metabolic dysfunction-associated steatotic liver disease and fibrosis. eGastroenterology 2, e100115. 10.1136/egastro-2024-100115 39872125 PMC11729351

[B57] WangD. LiM. LingJ. ChenS. ZhangQ. LiuZ. (2023). Assessing the effects of aging on the liver endothelial cell landscape using single-cell RNA sequencing. Hepatol. Commun. 7, e0021. 10.1097/HC9.0000000000000021 PMC989435236724124

[B58] Wang W. Qian J. Shang M. Qiao Y. Huang J. Gao X. (2024a). Integrative analysis of the transcriptome and metabolome reveals the importance of hepatokine FGF21 in liver aging. Genes and Dis. 11, 101161. 10.1016/j.gendis.2023.101161 PMC11252782 39022127

[B59] Wang W. Xu K. Shang M. Li X. Tong X. Liu Z. (2024b). The biological mechanism and emerging therapeutic interventions of liver aging. Int. J. Biol. Sci. 20, 280–295. 10.7150/ijbs.87679 PMC10750291 38164175

[B60] Watson C. J. E. Kosmoliaptsis V. Pley C. Randle L. Fear C. Crick K. (2018). Observations on the *ex situ* perfusion of livers for transplantation. Am. J. Transpl. 18, 2005–2020. 10.1111/ajt.14687 29419931 PMC6099221

[B61] Weissenbacher A. Bogensperger C. Oberhuber R. Meszaros A. Gasteiger S. Ulmer H. (2022). Perfusate enzymes and platelets indicate early allograft dysfunction after transplantation of normothermically preserved livers. Transplantation 106, 792–805. 10.1097/TP.0000000000003857 34144552

[B62] Wen Y. Ju C. (2025). New insights into liver injury and regeneration from single-cell transcriptomics. eGastroenterology 3, e100202. 10.1136/egastro-2025-100202 40735115 PMC12306295

[B63] Williams E. G. Pfister N. Roy S. Statzer C. Haverty J. Ingels J. (2022). Multiomic profiling of the liver across diets and age in a diverse mouse population. Cell Syst. 13, 43–57.e6. 10.1016/j.cels.2021.09.005 34666007 PMC8776606

[B64] Yang S. Liu C. Jiang M. Liu X. Geng L. Zhang Y. (2024). A single-nucleus transcriptomic atlas of primate liver aging uncovers the pro-senescence role of SREBP2 in hepatocytes. Protein and Cell 15, 98–120. 10.1093/procel/pwad039 37378670 PMC10833472

