# Supplementary material for: Multi-omics perspectives on mitochondrial vulnerability in the aging liver: biological modules and translational implications
Source: Front Cell Dev Biol. 2026 Oct 15;14:1954948. doi: 10.3389/fcell.2026.1954948 (PMC13716950; doi:10.3389/fcell.2026.1954948)
Supplement: Supplementary file 1 [file Table1.docx]

Supplementary Table S1

Representative evidence classified by biological context. The categories identify the dominant experimental setting and should not be interpreted as a formal quality score.

| **Representative study** | **Species/sample** | **Dominant context** | **Module(s)** | **Evidence boundary** |
| --- | --- | --- | --- | --- |
| Williams et al., 2022 | Genetically diverse mice | Physiological baseline | Lipid/metabolic; redox; proteostasis | Strong systems evidence; diet/genotype dependent |
| Nikopoulou et al., 2023 | Aged mouse liver | Physiological baseline | Lipid/metabolic; redox; spatial heterogeneity | Mouse tissue; no transplant outcome linkage |
| Huang et al., 2021 | Naturally aged male mice | Physiological baseline | Redox; mitochondrial quality; recovery | Sex- and model-specific |
| Duan et al., 2023 | Aged mice and mechanistic assays | Baseline plus mechanistic perturbation | Lipid/metabolic; redox | Specific 13-HODE-catalase mechanism; not a clinical biomarker |
| Maeso-Díaz et al., 2022 | Young and aged mice | Challenge model | Reduced recovery capacity | Mechanistic injury/recovery evidence |
| Wang et al., 2023; Liu et al., 2024, 2026 | Aged mouse liver/cells | Baseline and I/R challenge | Inflammatory sensitization | Predominantly mouse evidence |
| Puppala et al., 2023 | Female non-human primates | Physiological baseline | Proteostatic strain; ER stress | Preprint; no human validation |
| Sinha et al., 2026 | Mouse and human liver | Physiological baseline | Zonation; metabolic remodeling | Cross-species anchor; limited outcome data |
| Hautz et al., 2024 | Human donor livers during NMP | Machine perfusion | Stress response; graft quality | Translational association, not aging-specific |
| Dingfelder et al., 2025; Eden et al., 2025 | Human grafts during HOPE | Machine perfusion | Oxidative/mitochondrial injury | FMN linked to outcomes; protocol-specific |
| Pezzati et al., 2025 | Human grafts during NMP | Machine perfusion | Inflammatory sensitization | Small cohort; external validation required |
| Watson et al., 2018; Matton et al., 2019 | Human donor livers during NMP | Machine perfusion | Biliary/recovery capacity | Small cohorts and selected grafts |

Abbreviations: EAD, early allograft dysfunction; ER, endoplasmic reticulum; FMN, flavin mononucleotide; HOPE, hypothermic oxygenated perfusion; I/R, ischemia–reperfusion; NMP, normothermic machine perfusion.
